# Is the Development of Gestational Diabetes Associated With the ABO Blood Group/Rhesus Phenotype?

**DOI:** 10.3389/fendo.2022.916903

**Published:** 2022-06-22

**Authors:** M. Lemaitre, M. Passet, L. Ghesquière, C. Martin, E. Drumez, D. Subtil, A. Vambergue

**Affiliations:** ^1^ University of Medicine, Lille, France; ^2^ CHU Lille, Department of Diabetology, Endocrinology, Metabolism and Nutrition, Lille University Hospital, Lille, France; ^3^ Univ. Lille, CHU Lille, ULR 2694 - METRICS: évaluation des technologies de santé et des pratiques médicales, Lille, France; ^4^ CHU Lille, Department of Gynecology and Obstetrics, Lille University Hospital, Lille, France; ^5^ CHU Lille, Department of Biostatistics, Lille University Hospital, Lille, France; ^6^ European Genomic Institute for Diabetes, University School of Medicine, Lille, France

**Keywords:** gestational diabetes mellitus, pregnancy, ABO blood group, rhesus factor, risk factor

## Abstract

**Aims:**

There are few published data on the putative association between the ABO blood group/rhesus (Rh) factor and the risk of developing gestational diabetes mellitus (GDM). Our aim was to explore the link between each one factor and GDM development.

**Methods:**

All women having given birth at Lille University Medical Center (Lille, France) between August 1^st^, 2017, and February 28^th^, 2018, were tested for GDM, using the method recommended in the French national guidelines. The risk of GDM was assessed for each ABO blood group, each Rh phenotype and combinations thereof, using logistic regression models.

**Results:**

1194 women had at least one GDM risk factor. The percentage of GDM varied with the ABO group (p=0.013). Relative to group O women, group AB women were more likely to develop GDM (OR = 2.50, 95% CI [1.43 to 4.36], p=0.001). Compared with the Rh-positive O group, only the Rh-positive AB group had an elevated risk of developing GDM (OR = 3.02, 95% CI [1.69 to 5.39], p < 0.001).

**Conclusions:**

Our results showed that Rh-positive group AB women have a greater risk of GDM. With a view to preventing GDM, at-risk individuals could be identified by considering the ABO blood group phenotype either as a single risk factor or in combination with other risk factors.

## Introduction

The ABO blood group classification is based on the presence or absence of A and B antigens controlled by the gene coding for ABO glycosyltransferase (located on chromosome 9) (1). Increased susceptibility to many diseases have been linked to modulation of the expression of ABO blood group antigens, including infections (2), vascular diseases (3), and cancer (4). A few epidemiologic and genetic studies have examined possible associations between ABO blood and the risk of type 2 diabetes mellitus (T2DM); however, the results have been inconsistent. Group A was found to be associated with T2DM in some studies (5, 6), whereas group B protected against DM in others (7, 8). The rhesus(Rh)-negative group O and Rh-positive group A phenotype were significantly more frequent in a cohort of 224 diabetic patients in Nigeria than in controls (9). A prospective study of a cohort of 82,104 people in France found that group A and group B had a greater risk of T2DM, relative to group O (10).

Gestational diabetes mellitus (GDM) is defined as a glucose tolerance disorder with onset during pregnancy. In 2010, the Societé Francophone de Diabétologie/Collège National des Gynécologues et Obstétriciens français (SFD/CNGOF) have proposed a selective screening, based the preence of risk factors. The expert panel for the Franch guidelines has recommended GDM screening if at least on of the following convential criteria is present: maternel age ≥ 35 years, preconception BMI≥ 25 kg/m2, a personal history of GDM, or the presence of diabetes in a first degree relative, or birth of a child with macrosomia. It’s not excluded that other risk factor could be integrated in this screening’s strategy. Other risk factors such as polycystic ovary syndrome, metabolic syndrome have not however been retained in France. It is associated with elevated fetal-maternal morbidity and long-term complications in the mother and child. The incidence of GDM and pregestational diabetes is rising worldwide (11). It is generally accepted that women with GDM are at a greater risk of subsequently developing T2DM (12). Although glucose values usually normalize soon after delivery, underlying beta-cell dysfunction may persist.

In contrast to the data on T2DM, there are reports on the possible association between the ABO blood type and the risk of developing GDM. A study of 792 healthy Iranian women reported that AB individuals had significantly higher fasting glucose levels in the second trimester (13). Other larger studies have come to the opposite conclusion (14, 15). Even though the discrepancies between these studies might be due (at least in part) to genetic differences between ethnic groups, there is a need to investigate the possible relationship between the ABO/Rh phenotypes and GDM in other populations. Given the lack of robust literature data, the objective of the present study was to investigate this association in a large cohort of pregnant women in France.

## Research Design and Methods:

### Study Population

This single-center, retrospective observational study was conducted at Lille University Medical Center (Lille, France) and was based on electronic medical records that are routinely completed at delivery for every woman who gives birth. According to French law, patients are informed that care-related data may be used for research purposes unless he/she opposes this use. The present study data had been anonymized prior to analysis, and we registered the study database with the French National Data Protection Commission (*Commission nationale de l’informatique et des libertés* (Paris, France); reference: 21/846.

All patients who received antenatal care and gave birth at the Obstetrics and Gynecology Department at Jeanne de Flandres Hospital between August 1^st^, 2017, and February 28^th^, 2018, were tested for GDM, using the protocol recommended by the French-speaking Society of Diabetes and the French National College of Obstetricians and Gynecologists (16). The protocol involves the measurement of the fasting plasma glucose (FPG) level at the initial prenatal visit for women with one or more of the following risk factors: preconception body mass index (BMI), ≥25 kg/m^2^, age ≥35, a personal history of gestational diabetes, a child with macrosomia, or a familial history of diabetes. The expert consensus considers patients with fasting glucose ≥7 mmol/L at the initial visit to have type 2 diabetes, so the diagnostic criterion for GDM is FPG 5.1 to 6.9 mmol/L. Women with an initial FPG below 5.1 mmol/L were retested at between 24 and 28 weeks, using a 75 g 2-hour oral glucose tolerance test (OGTT); GDM was defined according to the criteria issued by the International Association of Diabetes and Pregnancy Study Group (17). The exclusion criterion were missing data, loss to follow-up, a lack of GDM screening, and other type of diabetes (i.e. women without GDM risk factors).

### Intervention

Once the diagnosis had been confirmed, the patients attended an initial consultation at which preventive hygiene and dietary measures were explained. The women were instructed to self-monitor their blood glucose six times a day (before and after each of their three meals). The results were collected using dedicated telemonitoring software (MyDiabby, Healthcare SAS, Bordeaux, France) and/or by phone with a specialist nurse twice a week. The women were given specific glycemic targets. Insulin therapy (either with short-acting insulin analogues before meals and/or long-acting insulin analogues at bedtime) was initiated when the glucose targets were not met after 7 to 10 days of good adherence to hygiene and dietary rules. The follow-up with an obstetrician complied with the French guidelines (16).

### Collected Data and Definitions

Data on the women’s demographic characteristics, the ABO blood group, the Rh phenotype and the presence of risk factors were extracted from medical charts. Data on age, preconception body mass index (BMI, in kg/m^2^), any previous pregnancies, and risk factors were collected from electronic and paper-based hospital records. GDM risk factors (including preconception BMI ≥25 kg/m^2^, age ≥35, a personal history of gestational diabetes, a child with macrosomia, and a family history of diabetes) were recorded. For patients with GDM, we also recorded the date of the GDM diagnosis, the type of GDM screening, the plasma glucose values (fasting or during an OGTT), the treatment start date, and the type of treatment (diet or insulin therapy). The management of gestational diabetes was to determine the proportion of women on dietary measures alone and the proportion on insulin.

### Laboratory Analysis

The ABO-RH blood group and the Rh-positive KEL 1 phenotype were determined at the French Blood Agency’s laboratory (Lille, France). The determination was based on automated hemagglutination in microplate assays (Qwalys, Diagast) or in column microfiltration assays (AutoVue Innova, Ortho or IH 500, Biorad).

### Statistical Analysis

Continuous variables are reported as mean (standard deviation, SD) when normally distributed or median (interquartile range, IQR) otherwise. Categorical variables are reported as frequency (percentage). The normality of distributions was assessed using histograms and using the Shapiro-Wilk test. Associations between maternal characteristics during pregnancy according to their ABO blood groups and rhesus were measured using analysis of variance for Gaussian continuous variables, Kruskal-Wallis test for non-Gaussian continuous variables and Chi-Square test for binary variables. The risk of having gestational diabetes was assessed for each ABO blood group, each rhesus system and combination thereof, using logistic regression models. Odds ratios (OR) and their 95% confidence intervals were reported as effect size. All statistical tests were done at the two-tailed α-level of 0.05 using the SAS software version 9.4 (SAS Institute, Cary, NC).

## Results

### The Study Population

**Figure 1:**
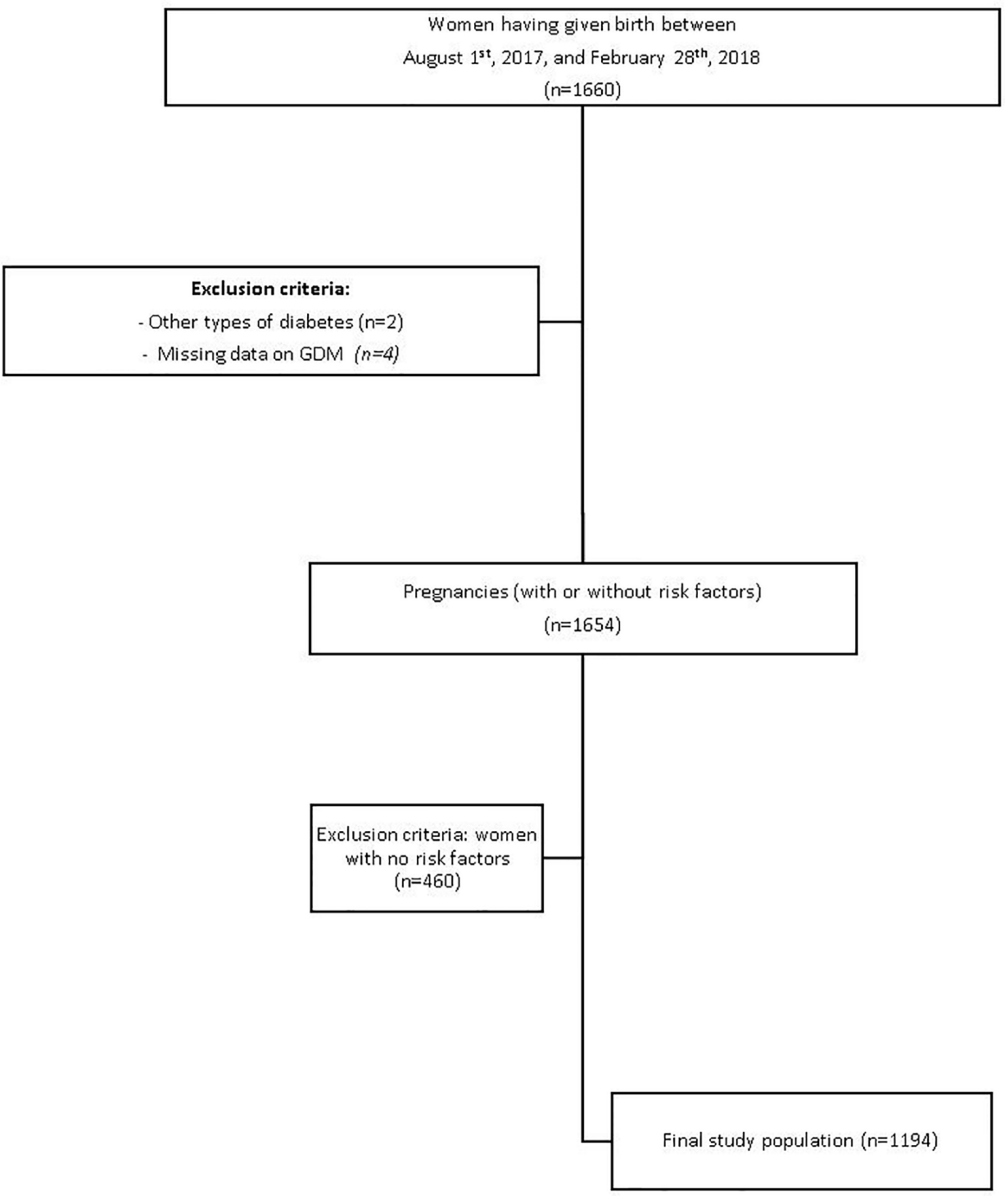
Study flow chart.

Between August 1^st^, 2017, and February 28^th^, 2018, 1660 women were screened for GDM and considered for enrollment in the study (**Figure 1**). We excluded 466 pregnancies: two women had T2DM before pregnancy, data on GDM status was missing for 4 patients, and 460 women had no GDM risk factors. Ultimately, we assessed 1194 women (351with GDM and at least one risk factor and 843 without GDM).

### Baseline Maternal Characteristics

The women’s clinical characteristics are summarized in **Table 1**. Overall, the mean age was 31.2 ± 5.8 years, and the median [IQR] preconception BMI was 26.2 kg/m^2^ [22.7 – 30.1]. Of the 1194 women, 429 (36.0%) had a first-degree family history of diabetes, 122 (10.3%) had a child with macrosomia, and 159 (13.4%) had a history of GDM. 34.2% of women with GDM and 33.1% without GDM had an age ≥35 years and respectively 70.1% and 57.8% have a BMI ≥ 25 kg/m^2^.With regard to the ABO blood group, there were 510 group O women (42.7%), 481 group A women (40.3%), 146 group B women (12.2%) and 57 group AB women (4.8%). 148 of the women (12.4%) were Rh-negative. After GDM screening, 351 (29.4%) women GDM, and 93 (26.8%) were being treated by insulin therapy and diet. The GDM group and non-GDM groups differed significantly with regard to the ABO blood group distribution but not with regard to the Rh phenotype.

**Table 1:** Baseline characteristics of the study population.

	Total (n = 1194)	With GDM (n=351)	Without GDM (n=843)
Age *years*	31.2 ± 5.8	31.3 ± 6.0	31.1 ± 5.7
Preconception BMI *kg/m^2^*	26.2 [22.7 ; 30.1]	27.5 [23.7 ; 32.1]	25.6 [22.3 ; 29.3]
1rst degree history of diabetes	429/1193 [36.0]	149/351 [42.5]	280/842 [33.3]
Personal history of macrosomia	122/1185 [10.3]	42/347 [12.1]	80/838 [9.5]
Personal history of GDM	159/1190 [13.4]	101/350 [28.9]	58/840 [6.9]
Nulliparity	418/1194 [35.0]	111/351 [31.6]	307/843 [36.4]
***ABO blood groups***			
Group O	510/1194 [42.7]	135/351 [38.5]	375/843 [44.5]
Group A	481/1194 [40.3]	145/351 [41.3]	336/843 [39.9]
Group B	146/1194 [12.2]	44/351 [12.5]	102/843 [12.1]
Group AB	57/1194 [4.8]	27/351 [7.7]	30/843 [3.6]
***Rhesus system***			
Rhesus -	148/1194 [12.4]	44/351 [12.5]	104/843 [12.3]
Rhesus +	1046/1194 [87.6]	307/351 [87.5]	739/843 [87.7]
GDM	351/1194 [29.4]	–	–
GDM with insulin therapy	93/347 [26.8]	93/347 [26.8]	–

Values are expressed as the number/total number (%), mean ± standard-deviation or median [interquartile range].

BMI, body mass index; GDM, gestational diabetes mellitus.

### Maternal Characteristics During Pregnancy, as a Function of the ABO and Rh Blood Groups

No differences were found in age or BMI as a function of the ABO blood group (**Table 2**). The first-trimester fasting plasma glucose differed significantly by ABO blood group (p=0.017) but not by Rh group (p=0.38).

**Table 2:** Characteristics of the study population during pregnancy, according to the ABO blood group and the Rh phenotype.

	O (n=510)	A (n=481)	B (n=146)	AB (n=57)	p	Rhesus - (n=148)	Rhesus + (n=1046)	p
Age *years*	31.2 ± 5.8 510/510	31.0 ± 5.9 481/481	31.1 ± 5.0 146/146	32.2 ± 6.0 57/57	0.56	30.4 ± 5.7 148/148	31.3 ± 5.8 1046/1046	0.087
BMI *kg/m^2^*	26.3 [22.6 ; 29.7] 504/510	26.0 [22.7 ; 30.1] 479/481	26.5 [22.9 ; 30.3] 145/146	27.3 [23.1 ; 31.6] 57/57	0.53	26.3 [22.0 ; 30.4] 148/148	26.2 [22.8 ; 29.9] 1037/1046	0.83
FPG 1^rst^ trimester *g/L*	4.73 ± 0.44 452/510	4.78 ± 0.45 425/481	4.71 ± 0.40 124/146	4.90 ± 0.51 50/57	**0.017**	4.79 ± 0.52 130/148	4.75 ± 0.43 921/1046	0.38
75 OGTT t.=0 min. *g/L*	4.46 [4.24 ; 4.73] 363/510	4.51 [4.24 ; 4.79] 339/481	4.46 [4.24 ; 4.79] 108/146	4.62 [4.46 ; 4.90] 39/57	**0.022**	4.51 [4.24 ; 4.79] 111/148	4.46 [4.24 ; 4.73] 738/1046	0.59
75 OGTT t.= 60 min. *g/L*	6.82 [5.67 ; 8.09] 353/510	7.10 [6.00 ; 8.31] 330/481	6.88 [5.67 ; 8.14] 102/146	8.11 [6.44 ; 9.24] 38/57	**0.009**	7.07 [5.72 ; 8.31] 106/148	6.93 [5.89 ; 8.20] 717/1046	0.74
75 OGTT t.= 120 min. *g/L*	6.05 [5.12 ; 7.15] 355/510	6.16 [5.28 ; 7.21] 333/481	6.35 [5.17 ; 7.21] 102/146	6.71 [6.00 ; 8.64] 39/57	**0.007**	6.44 [5.01 ; 7.54] 107/148	6.11 [5.23 ; 7.21] 722/1046	0.49
GDM	135/510 [26.5]	145/481 [30.1]	44/146 [30.1]	27/57 [47.4]	**0.013**	44/148 [29.7]	307/1046 [29.3]	0.92
GDM with insulin therapy	34/134 [25.4]	40/142 [28.2]	12/44 [27.3]	7/27 [25.9]	0.96	12/44 [27.3]	81/303 [26.7]	0.94

Values are expressed as the number/total number (%), mean ± standard-deviation, or median [interquartile range].

BMI, body mass index.

FPG, fasting plasma glucose.

OGTT, oral glucose tolerance test.

GDM, gestational diabetes mellitus. Significant values are in bold.

The blood glucose levels after 0, 60 and 120 minutes of the OGTT differed as a function of the ABO phenotype (p=0.022, p=0.009, p=0.007, respectively). The percentage of women with GDM differed significantly when comparing the ABO groups (p=0.013). No differences were found between Rh phenotype.

### Associations Between the ABO Blood Group and the Rh Phenotype With the Presence of GDM

**Figure 2:**
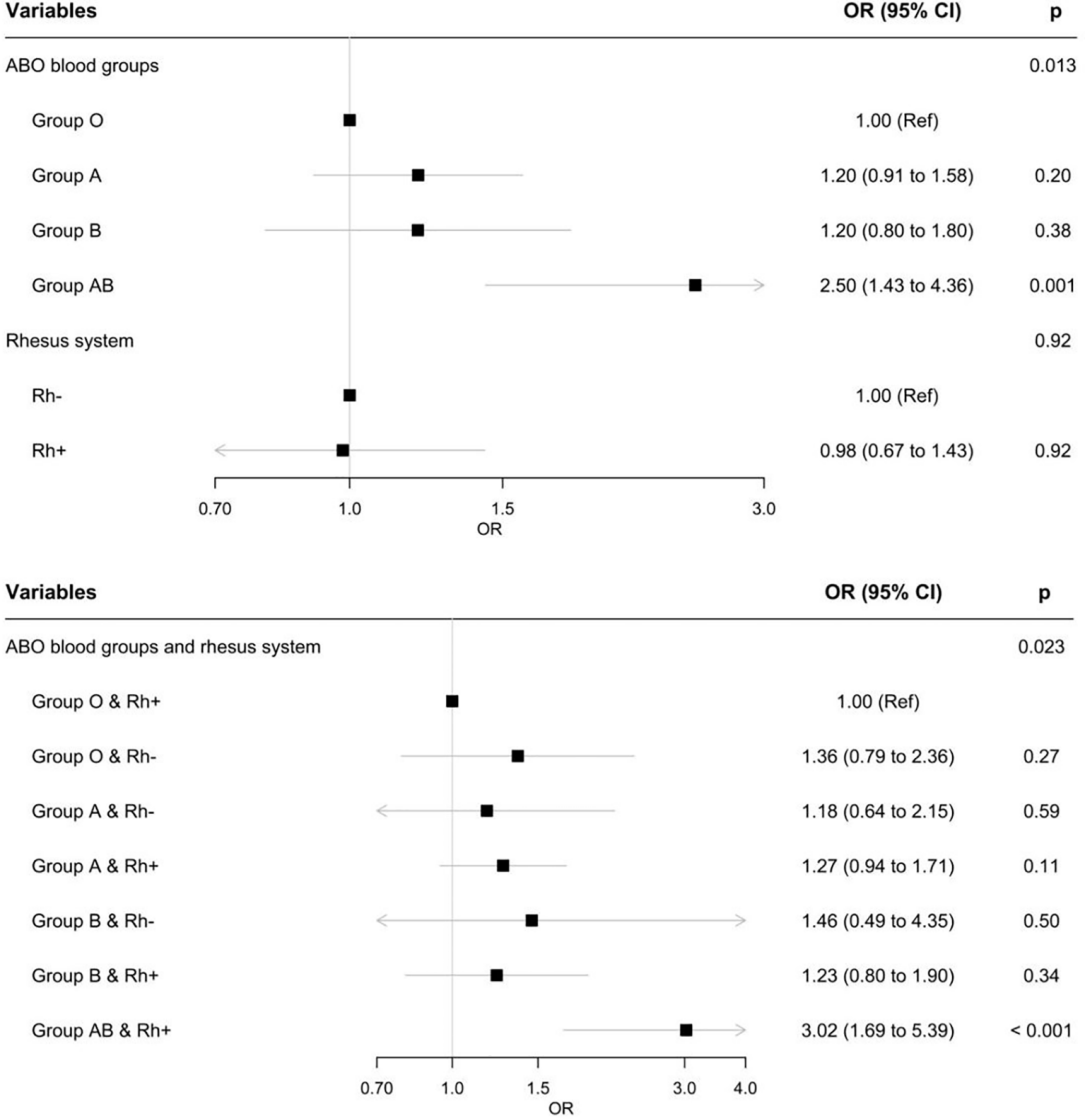
Associations between ABO blood groups and GDM, between the Rh phenotype and GDM, and combinations of ABO and Rh phenotypes and GDM.

Compared with group O, group AB women were more likely to develop GDM (OR=2.50 95%CI [1.43 to 4.36], p=0.001) (**Figure 2**). The differences were not statistically significant for blood groups A and B compared with group O (p=0.20, p=0.38, respectively).

One ABO/Rh combination were associated with the presence of GDM (p=0.023). Compared with Rh-positive group O women, Rh-positive group AB women had a significantly higher risk of developing GDM (OR =3.02,95%CI [1.69 to 5.39], p < 0.001). The analysis could not be performed for Rh-negative group AB women because the sample size (n=4) was too small.

## Discussion

Few researchers have examined the potential link between the ABO blood type or the Rh system and the development of GDM. The objective of the present single-center study was to determine whether a link was present among a relatively large cohort of women having given birth at a large French tertiary hospital. We found that the first-trimester fasting plasma glucose level varied significantly according to the ABO blood group (p=0.017) but not according to the Rh group. Interestingly, we also found that group AB women had a greater risk of developing GDM, relative to group O women (OR [95%CI] = 2.50, [1.43 to 4.36], p=0.001). Compared with Rh-positive group O women, only Rh-positive group AB had a significantly higher risk of developing GDM (OR [95%CI] = 3.02, [1.69 to 5.39], p < 0.001).

In the general population in France, the blood type distribution is 45% for group A, 9% for group B, 3% for group AB, and 43% for group O, 15% for the Rh-negative phenotype, and 85% for the Rh-positive phenotype (18). The blood type distribution in our study population (40.3%, 12.2%, 4.8% and 42.7% for groups A, B, AB and O, respectively) was therefore in line with the general population data. One can usually observe minor differences within countries and major differences between countries and continents; for example, the Rh-negative phenotype is extremely rare in Asia (19). Hence, caution must be taken when comparing our present results with data from non-French or non-European cohorts. Our GDM population cohort had much the same characteristics (in terms of age and BMI, etc.) as other French cohorts (20). The prevalence GDM (29.4%) is line with other studies performed in the Lille area, where the local population has a high prevalence of risk factors (overweight and/or obesity, a family history of diabetes, etc.).

A study of the French E3N cohort of 82104 patients found that the O blood group was associated with a lower risk of developing type 2 diabetes, relative to the A, B, and AB groups (10). However, the study publication did not report the percentage of women with a personal history of GDM (10). The latter results are consistent with a Nigerian study in which the proportion of people with an O+ group was significantly lower in patients with DM than in non-diabetics. In contrast to our present results, Nigerian people with O- or A+ blood groups appeared to be at a greater risk of developing DM in the (9). A study of a population in northwest Ethiopia suggested that antigen B was associated with a greater increased risk of T2DM, whereas a O blood group was associated with a lower risk (21).

The literature data on women with GDM are heterogeneous. A study of 792 pregnant women in Iran found that the second-trimester fasting blood glucose levels was higher in blood group AB women than in blood group A women (14). In a study of 233 women with GDM, Karagoz et al. found that the AB blood group was more frequent in patients with GDM than in the control group (p=0.029) (22). The disparities between these literature findings and our present results might be due to a difference in ABO blood group distribution in the population: the proportion of AB patients was greater in Karagoz et al.’s study (12% in the GDM group and 8% in controls) than in our study (7% and 3.8%, respectively). Furthermore, Karagoz et al.’s study did not report data on traditional risk factors for GDM. In contrast, risk factors were prevalent in our study population. Shimodaira et al. confirmed that the AB blood group was a risk factor for GDM in Japanese population having undergone universal, two-step screening for this disease (23). Here, we found that only the Rh-positive AB group had a significantly greater risk of developing GDM (OR = 3.02, 95% CI = 1.69 to 5.39, p < 0.001 vs. the Rh-positive O group). Shimodaira et al. could not adequately study the Rh phenotype because (as was mentioned above) Rh-negative status is extremely rare in Japan (0.5%) (23).

Our results differ from other published data. In a large, prospective, population-based study of pregnant women in China, the AB blood group was associated with a lower risk of GDM (relative to A, B, and O blood groups), and the largest group of non-GDM women had a B blood group (33.4%) (24). In contrast, the A blood group accounted for the largest group (45%) of non-GDM women in our study.

A retrospective cohort study in Israel found that the AB blood group was associated with a lower risk of developing GDM (defined according to Carpenter and Coustan’s criteria) than other blood groups, after adjustment for maternal age, parity, and the number of fetuses. The frequency of the Rh phenotype was similar in the GDM and control groups (25). Lastly, our findings are not in line with Sapanont et al.’s observations 600 pregnant women in Thailand who were screened for GDM screening according to Carpenter and Coustan’s criteria; in a regression analysis designed to adjust for traditional risk factors, the O blood group was independently associated with an elevated risk of GDM (26).

The Rh factor’s major roles are related to the membrane organization of phospholipids and the expression of various membrane glycoproteins. It has been suggested that the Rh factor can influence glucose transport and thus the development of diabetes. The few studies of Rh factor and GDM did not observe an association. In a Turkish study, the Rh-negative phenotype was significantly more frequent in diabetic patients than in control non-diabetic patients (27). Our study showed that only the Rh-positive AB women had a significantly greater risk of developing GDM. Our results therefore showed that in combination with other factors (i.e. blood groups), Rh-positive status increases the risk of GDM.

It is known that the ABO blood group distribution varies significantly from one ethnic group to another. Therefore, our results indicate that the strength of the association between the ABO blood group/Rh system and the risk of GDM will depend on the population in question. These results need certainly to be replicated in other popualtions. Even if this is a single center study, the implication is all women are French nationals. These findings might be quite a bit more generalizable to other western European population.

GDM is probably a multifactorial disease of pregnancy that can be induced by genetic factors, insulin resistance, and/or inflammatory processes. In view of the pathogenic similarity between T2DM and GDM, these biomarkers might be also involved in the pathogenesis of GDM. Identifying risk factors early in pregnancy might help to predict a subsequent clinical diagnosis of GDM. Our present results suggest that the incidence of GDM is higher for the AB blood group than for the other blood groups. Blood typing is an inexpensive test that could be readily performed during the antepartum period. Hence, the blood type might constitute another factor for predicting the occurrence of GDM. Accordingly, we suggest that the AB blood type could be added to the list of risk factors for GDM.

Our study had several strengths. Firstly, the present study was the first to address this topic in a population of women screened for GDM in accordance with the French national guidelines. Secondly, this was a large, population-based study in which all the traditional GDM risk factors were documented in detail. The study also had some limitations. Firstly, the single-center cohort design means that the data might not be readily generalizable - even though we checked that the ABO blood group distribution was similar to that of the general population in France. For France, the suggestion that AB blood type be considered as an additional risk factor would only apply for those who do not already have a risk factor, because those with other risk factor would be screened under existing guidelines. Unfortunately, patients without risk factor were excluded from the present study. Lastly, we did not access a number of variables though to influence with the development of GDM (e.g. the women’s levels of physical activity, socioeconomic factors, and weight gain during pregnancy).

## Conclusion

Our results showed that the AB/Rh-positive women have a higher risk of GDM. Given the clinical implications of GDM and the fact that ABO/Rh blood group phenotypes are stable over the lifespan, it is important to determine the nature of the association between the ABO blood groups and the risk of GDM. With a view to prevention and if our present findings can be replicated by studies of larger populations in other countries, it might be possible to use the ABO blood group phenotype (as a single risk factor or combined with other risk factors) to identify individuals at risk of GDM in early pregnancy. However, further epidemiological and genetic studies are needed to define the relationship between ABO blood groups and GDM. So, to date, the evidence for the relationship between ABO blood group and GDM is still limites and inconsistent. Even if Chen et al. will conducted a metaanalysis to further confirm the relationship between ABO blood group and GDM, it would be interesting to carried out others prospectives studies considering this risk factor alone or in combinaison with the others usuals risk factors in a Caucasian population (28).

## Data Availability Statement

The original contributions presented in the study are included in the article/supplementary material. Further inquiries can be directed to the corresponding author.

## Ethics Statement

The studies involving human participants were reviewed and approved by CNIL 21/846. The patients/participants provided their written informed consent to participate in this study.

## Author Contributions

MP collected data. ML and AV wrote the manuscript. CM, ED supervised and conducted the statistical analyses. LG, DS, CM, ED and AV reviewed the manuscript. AV initiated and supervised the study and reviewed the manuscript. AV is the guarantor of this work and, as such, had full access to all the data in the study and takes responsibility for the integrity of the data and the accuracy of the data analysis. All authors contributed to the article and approved the submitted version.

## Funding

This research did not receive any specific funding from agencies or organizations in the public, commercial, or not-for-profit sectors.

## Conflict of Interest

The authors declare that the research was conducted in the absence of any commercial or financial relationships that could be construed as a potential conflict of interest.

## Publisher’s Note

All claims expressed in this article are solely those of the authors and do not necessarily represent those of their affiliated organizations, or those of the publisher, the editors and the reviewers. Any product that may be evaluated in this article, or claim that may be made by its manufacturer, is not guaranteed or endorsed by the publisher.
